# The Effect of Ozone Treatment on the Physicochemical Properties and Biocompatibility of Electrospun Poly(ε)caprolactone Scaffolds

**DOI:** 10.3390/pharmaceutics13081288

**Published:** 2021-08-18

**Authors:** Lauryna Dabasinskaite, Edvinas Krugly, Odeta Baniukaitiene, Dainius Martuzevicius, Darius Ciuzas, Lina Jankauskaite, Lauryna Aukstikalne, Arvydas Usas

**Affiliations:** 1 Department of Environmental Technology, Kaunas University of Technology, LT-50254 Kaunas, Lithuania; edvinas.krugly@ktu.lt (E.K.); dainius.martuzevicius@ktu.lt (D.M.); darius.ciuzas@ktu.lt (D.C.); 2 Department of Polymer Chemistry and Technology, Kaunas University of Technology, LT-50254 Kaunas, Lithuania; odeta.baniukaitiene@ktu.lt; 3 Faculty of Medicine, Institute of Physiology and Pharmacology, Lithuanian University of Health Sciences, LT-44307 Kaunas, Lithuania; lin.jankauskaite@gmail.com (L.J.); grinlaur@gmail.com (L.A.); arvydas.usas@lsmuni.lt (A.U.)

**Keywords:** tissue engineering, electrospun scaffold, poly(ε)caprolactone, ozone treatment, hMDSC, IGF-1

## Abstract

Ozonation has been proved as a viable surface modification technique providing certain properties to the scaffolds that are essential in tissue engineering. However, the ozone (O_3_) treatment of PCL scaffolds in aqueous environments has not yet been presented. O_3_ treatment performed in aqueous environments is more effective compared with traditional, executed in ambient air treatment due to more abundant production of hydroxyl radicals (•OH) within the O_3_ reaction with water molecules. During interaction with •OH, the scaffold acquires functional groups which improve wettability properties and encapsulate growth factors. In this study, a poly(ε)caprolactone (PCL) scaffold was fabricated using solution electrospinning and was subsequently ozonated in a water reactor. The O_3_ treatment resulted in the expected occurrence of oxygen-containing functional groups, which improved scaffold wettability by almost 27% and enhanced cell proliferation for up to 14 days. The PCL scaffold was able to withhold 120 min of O_3_ treatment, maintaining fibrous morphology and mechanical properties.

## 1. Introduction

Tissue engineering provides conceptually new opportunities for regenerative medicine as an alternative to conventional surgical transplantation, which may suffer from immune and infectious responses [1,2]. It heavily relies on natural or synthetic biocompatible polymers serving as matrices (often referred to as scaffolds) imitating natural tissues [3,4].

Fibrous matrices have gained attention as scaffolds for tissue engineering due to their ability to influence cell migration, alignment, morphology, and function, thus mimicking the extracellular matrix (ECM) that effectively supports cell adhesion and proliferation [5,6]. In addition, such scaffolds favor the delivery of various growth factors that play an important role in regulation of cell fate. Most importantly, cells should be able to infiltrate the scaffold and support regenerative processes while facilitating gradual scaffold degradation [7]. Fibrous scaffolds can be produced using multiple techniques such as self-assembly, phase separation, and electrospinning [8,9]. Among these, electrospinning is considered as a very promising technique [10], providing high surface to volume ratio, interconnected pores, and fiber dimensions in the various controlled size ranges close to those of ECM-native structure [11,12].

Several parameters need to be considered when selecting a polymer for the fabrication of a fibrous scaffold, which is intended for regeneration of a damaged tissue. The scaffold-forming polymer should be biocompatible, non-immunogenic, and possess functional groups for attaching the active molecules/growth factors and their release in a controllable manner [13]. Moreover, it must support cell growth and be biodegradable and durable in order to ensure high regenerative potential [14]. Poly(lactic acid) (PLA), poly(glycolic acid) (PGA), poly(ε)caprolactone (PCL), and polyurethane (PU) have been applied for scaffold material production [3,15]. Among these, PCL has been reported as one of the most suitable and desired synthetic biodegradable polymers for production of scaffold materials with strong mechanical properties and biocompatibility, suitable for tissue engineering, drug delivery, and wound dressing [2,13].

Although PCL properties are highly attractive for tissue engineering, it also has several drawbacks, including low hydrophilicity and lack of functional groups necessary for incorporating growth factors [11,16]. Generally, growth factors are immobilized through the functionalized surface via physical (non-covalent), covalent, and bioaffinity reactions [17,18]. The growth factor (GF) absorption to the scaffold is determined by the following parameters: wettability, surface area, and electrostatic attraction. Therefore, the surface of the scaffold is often functionalized by modifying the fiber surface chemically and physically, or by incorporating bioactive molecules [19]. A variety of methods are being used to modify scaffolds for tissue engineering, such as treatment with plasma/ozone, peroxides, sodium hydroxide (wet-chemical method), surface graft polymerization, electrospinning, etc. [5,20,21,22]. The selection of the modification method is determined according to the nature of the scaffold material and possible chemical bonds that can be established, avoiding deterioration of mechanical and morphological properties of the scaffold. The modification not only changes the chemical composition of the surface, but also improves hydrophilic properties, providing a more favorable environment for cellular adhesion [5]. In case of electrospinning, the sensitive molecules, therapeutic agents, and cells should be incorporated after all treatments aiming to protect them from additional physicochemical stresses [23].

Ozone (O_3_) treatment is a convenient, easy, and cost-effective method. Initially, it has been employed to sterilize heat-sensitive materials against a variety of microorganisms, but an improvement in hydrophilicity was observed [24] due to the introduction of chemically active groups on the fiber surface, such as carboxyl and hydroxyl groups [25]. The treatment may be conducted in both gaseous and aqueous phases. The latter is more efficient compared with the air environment, since ample availability of water molecules results in a higher amount of non-selective free hydroxyl radicals (•OH), which possesses the highest reactivity of reactive oxygen species [25,26]. Following such treatment, fibers enhance cell adhesion and proliferation without any significant changes in morphology of scaffold fibers [27,28]. At the same time, selecting an optimum treatment time is very important, since prolonged treatment may result in decreased mechanical and chemical properties of a scaffold, while cell proliferation may remain unaltered or even improved [27]. Yet, there is a lack of studies reporting the changes in physicochemical and biological properties of ozone-treated PCL scaffolds.

The aim of this study was to research surface functionalization of electrospun PCL scaffolds under aqueous O_3_ treatment, aiming to enhance the incorporation of growth factors and support cell growth. The study contributed to the further development and applications of PCL scaffolds and their surface modification for tissue engineering applications.

## 2. Materials and Methods

### 2.1. Materials

Polycaprolactone (PCL, IUPAC name: (1,7)-Polyoxepan-2-one, CAS: 24980-41-4, Mn ~80 kDa, Cat.No: 440744, St. Louis, Missouri, USA), Toluidine Blue O (TBO, Mw: 305.83 g/mol, CAS: 92-31-9, St. Louis, Missouri, USA), Dulbecco’s Phosphate Buffered Saline (PBS, Lot No. RNBH1585, St. Louis, Missouri, USA), Sodium dodecyl sulfate (SDS, ≥98.5%, CAS: 151-21-3, St. Louis, Missouri, USA), and aluminum foil (Lot No. 515981, St. Louis, Missouri, USA) were purchased from Sigma-Aldrich Corp., St. Louis, Missouri, USA. Acetone (HPLC grade, CAS: 67-64-1), N.N-Dimethylformamide (DMF, HPLC grade, CAS: 68-12-2) and Sodium hydroxide solution (NaOH, 0.1 N, CAS N 1310-73-2) were purchased from Eurochemicals (Spa, Milan, Italy).

### 2.2. Design of Experiment, Data Analysis and Quality Control

The study was fulfilled in four stages, as explicitly presented in the following subchapters:

1. The fabrication of the PCL scaffold by electrospinning;
2. Surface modification of the scaffold by O_3_;
3. Fixing of the Insulin-like growth factor;
4. Characterization of the performance of the fabricated scaffold.

Each sample was prepared and analyzed in triplicates. The differences between samples were assessed using a two-sample T-test implemented in a data analysis package (v2019, OriginLab Corporation, Northampton, MA, USA). Statistical significance was accepted at *p* < 0.05.

Analysis of images obtained by SEM was performed using image processing software (ImageJ, NIH, Bethesda, MD, USA) by dividing the image into four equal quartiles and selecting all points on one quartile. Data were expressed as mean ± standard deviation or median and 1.5 interquartile range interval.

### 2.3. Fabrication of Polymer Scaffold

Polymer solutions (10–25% *w*/*v*) were prepared by dissolving PCL pellets in 2:3 (*v*/*v*) acetone and N.N-Dimethylformamide mixture (Table 1). Investigation of PCL concentration in the spinning solution was considered as the main variable in the scaffold fabrication experiment, and thus other parameters of electrospinning process (such as voltage, polymer supply rate, tip-to-collector distance) were slightly adjusted to achieve a stable polymer jet formation. The mixing process was carried out at 40 °C on a magnetic stirrer at 200 rpm (LBX H03D series, 3 L, IBX Instruments, Barcelona, Spain).

**Figure 1:**
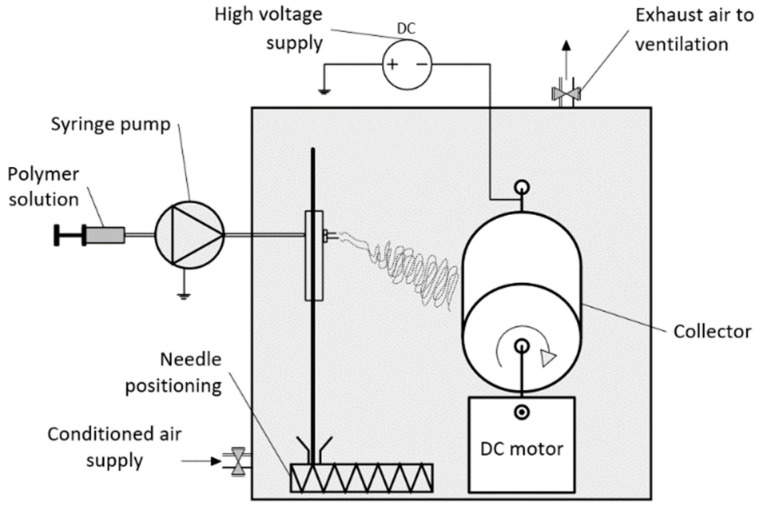
Electrospinning setup.

An in-house solution electrospinning setup (Figure 1) was designed and manufactured at the Kaunas University of Technology, Lithuania. The polymer solution was loaded into a 10 mL plastic Luer lock syringe (B. Braun, Bethlehem, Pennsylvania, USA) equipped with a blunt 21-gauge steel needle (Fisnar, Germantown, Wisconsin, USA). Flow rates from 2.1 mL/h to 2.3 mL/h were modulated using a syringe pump (RobotDigg XK-syringe-Full, Shanghai, China). The needle was connected to a power supply and directed towards a grounded rotating metal drum collector (rotation speed 16 rpm). The tip-to collector distance ranged from 11 to 14 cm. High voltage was modulated by an in-house high voltage supply (dual positive DC 0-50 kV) up to 22 kV. Each sample was electrospun using two syringes simultaneously formed a sample from, at total, ~20 mL of polymer solution. An IR lamp was used to heat the solution to avoid polymer coagulation in the syringe and connecting tubes. The temperature (T, °C) and relative humidity (RH, %) inside of the electrospinning chamber were controlled within 30 ± 2 °C (T) and 40 ± 2% (RH). After electrospinning, the electrospun mats were stored in a vacuum chamber at 21 ± 2 °C for a period of 12 h.

### 2.4. Scaffold Surface Modification

The treatment may be conducted in both gaseous and aqueous phases. The latter is more efficient compared with the air environment, since ample availability of water molecules results in a higher amount of non-selective free hydroxyl radicals. Free hydroxyl radicals (•OH) react with PCL and break the polymeric chain of PCL to form oligomers and monomers having hydroxyl and carboxyl functional groups, which exist in hydrogen bonding with excess water:

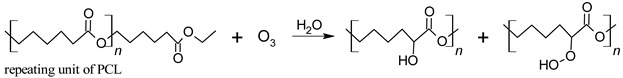
(1)

Fabricated samples were cut into pieces of 50 mg and treated with O_3_ in water using an O_3_ generator (GL-3188A, Shenzhen Guanglei, PRC) with an output of 400 mg/h. Samples were placed in a round-bottomed flask filled with 150 mL of deionized water. The treatment was performed at room temperature (20 ± 1 °C) for the period of 30, 60, 120, and 150 min. After that, samples were again stored in a vacuum dryer at 21 ± 2 °C for 12 h. Since the samples where thin enough, the pore sizes and interconnectivity were sufficient, and the ozone treatment was applied efficiently. Otherwise, if the pores and interconnectivity is lacking, a few drops of >70% ethanol can be added to ensure better diffusion.

### 2.5. Immobilization and Release Kinetics of Growth Factor

Insulin-like growth factor-1 (IGF-1) was selected during this research due to its ability to promote stem cell proliferation and differentiation to neurogenic [29], osteogenic [30], and chondrogenic [31]. Furthermore, IGF-1 can be used for the treatment of various tissues, including muscle, bone, cartilage, liver, lung, nerve, and etc. [18,32].

Recombinant human Insulin-like growth factor 1 (IGF-1, Life Technologies, Carlsbad, California, USA) was obtained in freeze-dried form and was used without further purification. The vial with IGF-1 was reconstituted according to the manufacturer’s instructions. IGF-1 was immobilized within the PCL scaffold by directly incorporating 1 µg of growth factor per 6 mm diameter of scaffold. The amount of released IGF-1 was quantified using a human IGF1 Elisa kit (ab100545, Abcam plc, Cambridge, UK) according to the manufacturer’s instructions. In brief, samples and standards were added to the antibody (specific for human IGF-1)-coated wells. All of the wells were washed after incubation, and then biotinylated antibody was added. HRP-conjugated streptavidin was added to the wells after the unbounded biotinylated antibody was washed away. The wells were again washed and a TMB substrate solution was added. When the solution changed color from blue to yellow, the intensity was measured at a wavelength of 450 nm (Multiskan GO 1.00.40, Thermo Fisher Scientific, Waltham, MA, USA).

IGF-1 release profile data were used to analyze the release kinetics by fitting them to four mathematical models: the zero-order, first-order, Hixson–Crowell, and Higuchi [33,34]. In order to determine the best fit, coefficient of determination (R^2^) values were generated using simple linear regression. Best fitted model was used to calculate the release constants (k) for different treatment duration IGF-1 release profiles. According to the k values, the rates of IGF-1 release from scaffolds were then compared.

### 2.6. Physicochemical Characterization

#### 2.6.1. Morphology

The surfaces of the scaffolds were analyzed using scanning electron microscopy (SEM S-3400N, Hitachi, Krefeld, Germany).

#### 2.6.2. Chemical Composition

The potential changes in scaffold chemical composition were screened by recording attenuated total reflectance, the Fourier transform-infrared (FTIR) spectra (Perkin-Elmer Frontier, Waltham, MA, USA) of the scaffolds. All spectra were recorded in the range from 4000 to 650 cm^−1^.

#### 2.6.3. Carboxyl Groups

The number of carboxyl groups introduced on the surface of the PCL scaffold was determined by Toluidine Blue (TBO) assay [35,36]. In brief, 0.1% of TBO solution was prepared by dissolving the dye in 1 mM NaOH. The scaffold (50 mg) was incubated in 3 mL of TBO solution for 24 h at 37 °C. The unbound dye was removed by washing the scaffold with 1 mM NaOH until the washing solution appeared clear. Attached TBO dye was then removed by immersing the scaffold in 3 mL 20% Sodium dodecyl sulfate (SDS) solution for 24 h. Once the dye was desorbed from the surface, the light absorbance of the solution was measured at 625 nm. The number of carboxyl groups on the scaffold surface was calculated from a calibration curve of TBO standard solutions (5, 10, 20, 30, 40, and 50 µM). The calculations were performed with the assumption that carboxyl groups interact with TBO stoichiometrically.

#### 2.6.4. Hydrophilicity

Water contact angle (θ) was measured to estimate the hydrophilicity/hydrophobicity of the scaffolds with an optical tensiometer Theta Lite TL 101 (Biolin Scientific, Espoo, Finland). A 20 µL droplet of distilled water was poured on the scaffold surface at room temperature (20 ± 1 °C) and the contact angle was measured by the included software (OneAttension v1.0, Biolin Scientific).

#### 2.6.5. Absorption Capacity

Absorption of liquid capacity (ALC) of the prepared scaffolds was determined in a phosphate-buffer solution (PBS, pH 7.4) at 37 °C. The samples were cut into 6 mm diameter discs, weighed, and placed in Eppendorf plates filled with 5 mL of PBS. The samples were removed after the predetermined time interval, carefully wiped with a filter paper, and then weighed.

ALC was calculated using the Equation (2):

ALC (%) = (W_w_ − W_d_)/W_d_ ∗ 100 (2)

where W_w_ and W_d_ represent the weights of the wet and dry sample, respectively.

#### 2.6.6. Crystallinity

X-ray diffraction analysis (XRD) of the samples was carried out with diffractometer BRUKER AXS D8 ADVANCE (BRUKER AXS, Karlsruhe, Germany) using Ni-filtered Cu Kα radiation. The detector moving rate was 0.02°, the intensity measurement was 0.5 s, the anode voltage Ua was 40 kV, and the current I was 40 mA. The accuracy of XRD analysis was 2θ = 0.01°.

Crystallinity degree (CI, %) was calculated using the Equation (3):

(3) $$CI=\frac{A_{1}}{A_{2}}\times 100$$

where: *A*_1_—area of crystalline peaks; *A*_2_—area of all peaks. Area of crystalline peaks was calculated using OriginPro (OriginLab Corporation, Northampton, MA, USA) and Excel (Microsoft Corporation, Redmond, WA, USA) software.

#### 2.6.7. Thermal Properties

Thermogravimetric analysis was performed using a TGA 4000 (Perkin Elmer, Waltham, MA, USA) thermal analyzer. The experiment was carried out under a nitrogen atmosphere, scanning from 34 up to 600 °C with a rate of 10 °C min^−1^.

Differential scanning calorimetry (DSC) was performed using thermal analyzer (Perkin Elmer DSC 8500, JAV). The experiment was performed under a nitrogen atmosphere in an aluminum crucible at a heating rate of 5 °C min^−1^ starting from 0 up to 250 °C.

#### 2.6.8. Mechanical Properties

The mechanical properties (Young’s modulus (E, MPa)) were determined using a universal material testing machine Zwick/Roell BDO-FB O.5 TH (Zwick, GmbH & Co, Ulm, Germany). Samples for mechanical testing were cut out from each fiber matt in a rectangular shape of 60 mm × 15 mm.

### 2.7. In Vitro Testing

#### 2.7.1. hMDSC Expansion and Seeding

Human muscle-derived stem cells (hMDSC) were obtained from 35-year-old female skeletal muscle (protocol No G2-133, approved by the regional Bioethics Committee). hMDSC were isolated and characterized as previously described [37,38]. Cells were cultured in Dulbecco’s Modified Eagle’s Medium (DMEM, Gibco, Paisley, UK) (4.5 g glucose/L) supplemented with fetal bovine serum (10% *v*/*v*) (FBS, Gibco, Paisley, UK), horse serum (10% *v*/*v*) (HS, Gibco, Paisley, UK), chicken embryo extract (0.5% *v*/*v*) (CEE, LSP, UK), penicillin (100 UI/mL), and streptomycin (100 µg/mL) (Penicillin/Streptomycin (10,000 U/L), Gibco, Paisley, UK). hMDSC were maintained at 37 °C in 5% CO_2_ in a humidified incubator. Media were changed every 2–3 days. For passaging, cells were twice washed with PBS and then detached and singularized with TrypsinLE Express (Gibco, UK). Passages 2–5 were used for further experiments. Cells were seeded on PCL and PCL/IGF-1 scaffolds (30,000 cells/scaffold) and cell-scaffold constructs were cultured in 96-well plates with culture medium at 37 °C in 5% CO_2_ and analyzed at day 1 (24 h), day 3, day 7, and day 14 of culture. Cells seeded on the plastic surface of the well in 96-well plates were used as control.

#### 2.7.2. Proliferation Assay

A proliferation assay was performed using a cell-counting kit (CCK-8, Dojindo Molecular Technologies, Inc., Tabaru, Japan) according to the manufacturer’s instructions. Briefly, 100 μL CCK8 reagent and cell culture medium (CM) (*v*/*v* 1:10) was added to each well in the 96-well plate with cell-scaffold constructs or control cells and incubated for 4 h at 37 °C. The absorbance at 450 nm was measured. Proliferation assays were performed at day 1, day 3, day 7, and day 14.

## 3. Results and Discussion

### 3.1. Morphology

**Figure 2:**
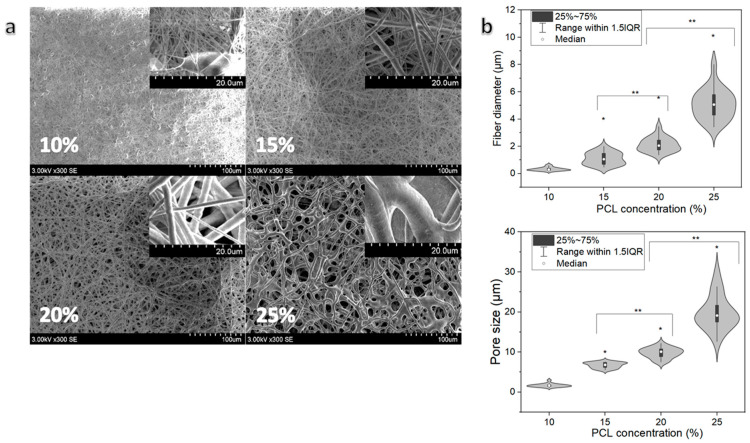
Morphology of electrospun PCL samples. (**a**) SEM micrographs of scaffolds obtained from solutions having PCL concentrations: 10%; 15%; 20%; 25%; (**b**) PCL fiber diameter and pore size distributions, (*) indicates statistically significant differences compared with the untreated group (0 min), (**) indicates statistically significant differences between groups, *p* < 0.05.

The fabricated PCL scaffolds featured a characteristic fibrous structure with randomly oriented fibers (Figure 2a). Such a design has been recognized as beneficial for cells, resulting in higher viability compared to aligned fibers [39,40]. The PCL concentration had a direct positive effect to the fiber diameter and pore size. Thinner fibers (median of 0.3 µm) were observed in the case of the 10% PCL sample (Figure 2a). Small pore size (Median 1.6 µm) was also observed in the 10% PCL sample (Figure 2b). At higher concentrations, the average fiber diameter and pore size increased significantly by an order of magnitude (median 5.1 µm and 19.0 µm, respectively, Figure 2b). The electrospinning of 10% PCL solution resulted in the formation of beaded fibers (Figure 2a) due to a low viscosity of the solution, as indicated in earlier studies [41]. At the other end of the tested concentration range of 25%, the electrospinning process was not stable, and the fiber morphology was not uniform, i.e., the fibers were fused to each other compared with fiber samples with lower concentrations (Figure 2a). All results were significantly different to each other, indicating the significance of changes in concentration. The most uniform morphology and stable electrospinning process was observed with samples containing 15% and 20% PCL. The 20% sample was chosen for further investigations due to a thicker fiber (2.1 µm) and pore size (10.1 µm) compared with the 15% sample (1.1 µm and 6.8 µm, respectively).

**Figure 3:**
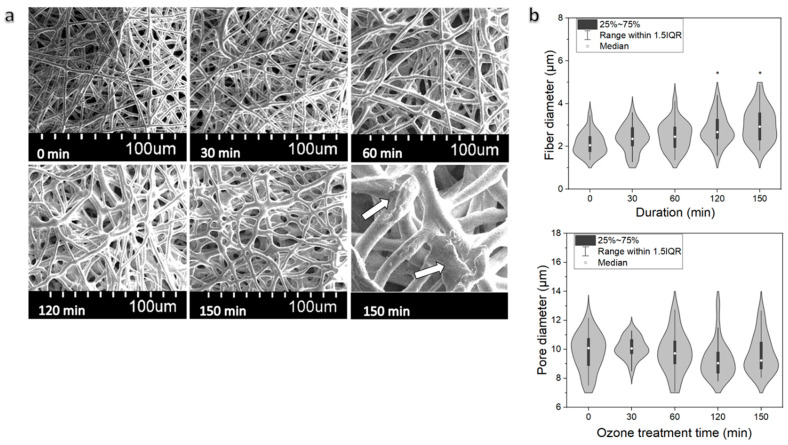
The effects of O_3_ treatment duration on (**a**) PCL scaffold morphology; (**b**) fiber diameter and pore size distribution, (*) indicates statistically significant difference compared with the untreated group (0 min), *p* < 0.05.

The 20% PCL scaffold was further treated with O_3_ in a water environment. The ozonation did not seem to have any substantial effect to fiber consistency for up to 120 min. However, after 120 min, partly fractured fiber areas emerged, indicating damage to the polymer structure (Figure 3a). There was no significant difference observed in pore size (median from 9.0 to 10.1 µm, compared to 10.1 after 0 min treatment), although fiber diameters were affected when treatment time was 120 min or more, i.e., the median fiber diameters were 2.7 µm and 2.9 µm, (120, 150 min) compared with 2.1 µm of untreated scaffold (Figure 3b).

### 3.2. Chemical Properties of PCL Scaffolds

**Figure 4:**
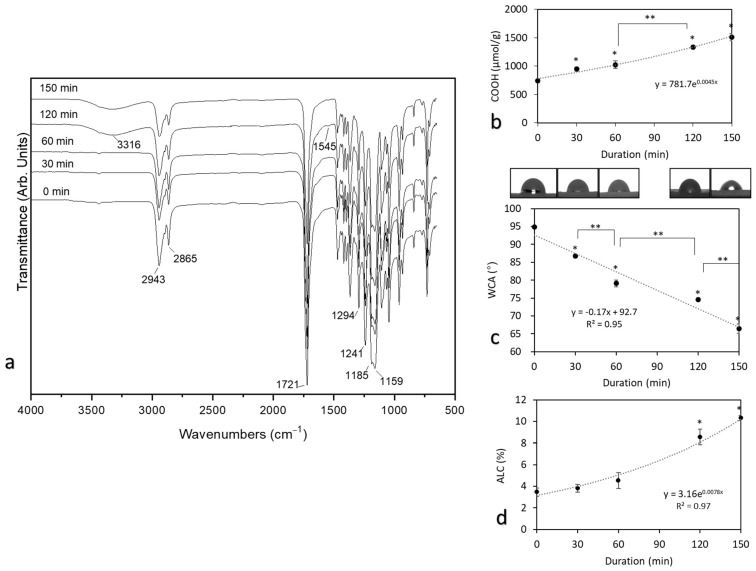
The effects of the treatment of the PCL scaffold by O_3_ after 0, 30, 60, 120, and 150 min: (**a**) ATR-FTIR spectrum (**b**) amount of carboxyl groups, (**c**) water contact angle (WCA), (**d**) absorption of liquid capacity (ALC), (*) indicates a statistically significant difference compared with the untreated group (0 min), (**) indicates a statistically significant difference between groups, *p* < 0.05.

The ATR-FTIR spectra (Figure 4a) of untreated and O_3_-treated scaffolds featured typical peaks for PCL:

- 2943 cm^−1^ asymmetric CH_2_ stretching;
- 2865 cm^−1^ symmetric CH_2_ stretching;
- 1721 cm^−1^ carbonyl (C=O) stretching;
- 1294 cm^−1^ C–O and C–C stretching in the crystalline phase;
- 1159 cm^−1^ C–O and C–C stretching in the amorphous phase;
- 1241 cm^−1^ asymmetric C–O–C stretching;
- 1185 cm^−1^ OC–O stretching. Such spectra have been registered in earlier studies [42].

An observable difference between FTIR spectra (Figure 4a) appeared after prolonged O_3_ treatment (starting at 120 min and continuing to 150 min). The band at 1294 cm^−1^ was associated with the stretching vibrations of C–O and C–C in the crystalline phase of PCL. The decrease of the intensity of these bands after ozone treatment may suggest that the crystallinity decreases after treatment. Broadband at 1190–1160 cm^−1^ is associated with the symmetric C–O–C stretching, as well as stretching vibrations of C–O and C–C bonds in the amorphous phase of PCL [43]. The peak intensity of these bonds increased as the treatment time was prolonged, suggesting that the samples became more amorphous. The broad absorption band in the 3500–3200 cm^−1^ range appeared, which was assigned to the stretching vibration of the OH group. The peak in the range of 1500–1600 cm^−1^ appeared due to the carboxyl functional group [25]. Earlier studies that used UV/O_3_ treatment have also reported similar absorption peaks, indicating surface modification [25,28]. On the other hand, treatment with O_3_ in the gaseous phase was shown to not result in OH peaks [27], meaning that our treatment method was more efficient, although we did not use UV as the addition of the treatment. Since molecular O_3_ reacts with water molecules via a chain reaction mechanism, it produces free hydroxyl radicals (•OH). •OH is an oxidant stronger than molecular O_3_ and reacts with the construct non-selectively, also meaning that O_3_ treatment in a water environment is more efficient [44].

The increased amount of carboxyl groups (COOH) was directly associated with the prolonged O_3_ exposure duration; 30 min treatment already introduced a significantly higher amount of carboxyl groups, while 120 min provided another significant increase from 60 min (Figure 4b). The amount of carboxyl groups doubled from 744 ± 43.2 (untreated) to 1510 ± 85.7 µmol/g (150 min), which is 103% accession in total. This indicates that the ozonation of electrospun PCL constructs in aqueous environments was very efficient compared with earlier attempts, i.e., 349 nmol/g (total increase—23%) as reported by Samsudin et al. (2018) [28], who subjected PCL microcarrier constructs to gaseous O_3_. This significant difference appeared due to the different treatment environment, as well as our improvement in the TBO assay. Since PCL is a hydrophobic polymer, we upgraded the COOH detection technique by increasing the incubation time to 24 h in total, while Samsudin et al. (2018) only used a duration of 30 min, which is not sufficient for the full penetration of the TBO dye. Carboxylation was confirmed by the broadening of FTIR peaks in the region of 2500–3300 cm^−1^ on the samples due to the OH bond stretching of the COOH group. These results support the TBO analysis results, i.e., that the hydrophilicity of the modified PCL increases with the carboxyl group density on the surface [45]. Furthermore, Sahoo et al. claimed that hydrogen bonding has a significant influence on the peak shape and intensities, generally causing peak broadening and shifts in absorption to lower frequencies [46]. Due to these reasons written above, the FTIR-ATR method is typically used to identify the functional groups rather than performing quantitative measurement where TBO usually is used. Other researchers also found that while it is hard to see the quantity of COOH groups by the FTIR peaks, the TBO analysis serves well in calculating the concentration of these groups [47].

As expected, incorporated oxygen-containing functional groups improved the hydrophilicity of the scaffolds. The WCA value reduced from 95 ± 0.4° (unmodified scaffold) to a value 66 ± 1.2° (150 min, Figure 4c), which comprised a 26% decrease. This was a slightly higher improvement compared with Samsudin et al. (2017), who investigated the WCA of the PCL microcarrier after UV/O_3_ treatment and managed to reduce the overall WCA value by ~5% [42]. There are publications that claim that the best cell attachment and proliferation was observed when the WCA angle was in the range from 40 to 70 [48,49], while other sources claim that it is enough for the surface to be hydrophilic (>90) [50,51]. However, not only the wettability affects the proliferation of cells. Factors like surface topography or adhesion, fiber size, pore size, interconnectivity, and etc. are also important and play a major role in overall proliferation [52,53,54].

Absorption of liquid capacity (ALC) is another estimate providing insights to the variations of hydrophilicity of a scaffold followed by O_3_ treatment (Figure 4d). The absorption of PBS ranged from 3.5 ± 0.4% (unmodified scaffold) to 10.3 ± 0.2% (150 min), suggesting that the absorption capacity of O_3_-treated scaffolds increased threefold, indicating an effective modification of PCL scaffolds. This correlated well with the quantity of carboxyl groups, which showed a significant increase after 120 min of treatment, and only the shift in ALC was more pronounced.

**Figure 5:**
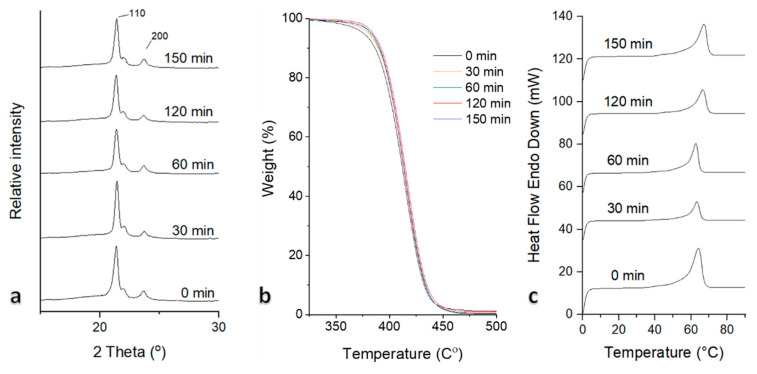
XRD (**a**), TGA (**b**), and DSC (**c**) plots of PCL scaffold with O_3_ treatment duration of 0, 30, 60, 120, and 150 min. Thermal analysis (by TGA and DSC) was performed to investigate the thermal properties of electrospun PCL samples. Although the testing range of such analyses do not represent the human tissue environment of 42 °C, they allow for understanding thermodynamic properties that occurred during the physic-chemical transformations induced by heating [57,58]. The thermal decomposition of all scaffolds was similar and occurred in a single step (Figure 5b), ranging from approximately 370 °C to 450 °C. No significant difference in weight reduction was registered for the tested O_3_-treated samples.

The scaffolds maintained the semicrystalline nature of the PCL polymer [55], as indicated by the typical distinct PCL peaks in XRD spectra at Bragg angles 2θ = 21.3° and 2θ = 23.6° (Figure 5a). No peak shifts were observed while comparing O_3_-treated samples against untreated, indicating similar crystal orientation in all PCL samples; 2θ = 21.3° and 2θ = 23.6° correspond to (110) and (200) crystallographic planes, respectively. The intensity of (110) and (200) planes was much weaker as the O_3_ treatment time was increased followed by the decrease of the crystallinity degree from 56.7% to 48.6% (after 0 min and 150 min treatment). For samples that were treated for 30, 60, and 120 min, the CI values were 55.8, 55.6, and 54.4% respectively, which suggests that O_3_ treatment reduces the crystallinity of electrospun PCL, turning it to more amorphous structure. Such a phase shift allows predicting the biodegradability of PCL. Typically, PCL is synthetic biodegradable and semi-crystalline polymer, whose surface starts degrading upon exposure to water. Due to hydrolysis, the amorphous regions begin to degrade followed by the crystalline ones. PCL scaffolds became more amorphous after treatment; therefore, the biodegradation process accelerated compared with untreated scaffolds. Since growth factors are connected to the scaffold within active functional groups that appear after treatment, breakage of polymer chains may lead to the release of the incorporated growth factors from the scaffolds by diffusion [56].

The temperature at the top of the peak in DSC plot is usually considered as the polymer’s melting temperature, followed by the endothermic transition to the molten state [59]. A slight variation in the melting point was observed among the samples (Figure 5c). The untreated sample melted at 64.1 °C. (0 min, Figure 5c). The 60 min-treated scaffold exhibited the lowest melting point of 62.8 °C, but the increasing treatment duration above 60 min resulted in an increased melting point to 66.6 °C (120 min, Figure 5c) and 67.5 °C (150 min, Figure 5c). Significant differences occurred only when the treatment time was 120 min or greater, indicating that degree of crystallinity was decreased. An increase in the melting point corresponded to a decrease in crystallinity of the polymer [60]. Our findings differed from those previously reported, i.e., Rediguieri et al. (2017), who did not find any significant changes following treatment duration [27], and this could be related to different O_3_ treatment environments compared with ours.

### 3.3. Mechanical Properties of PCL Scaffolds

**Figure 6:**
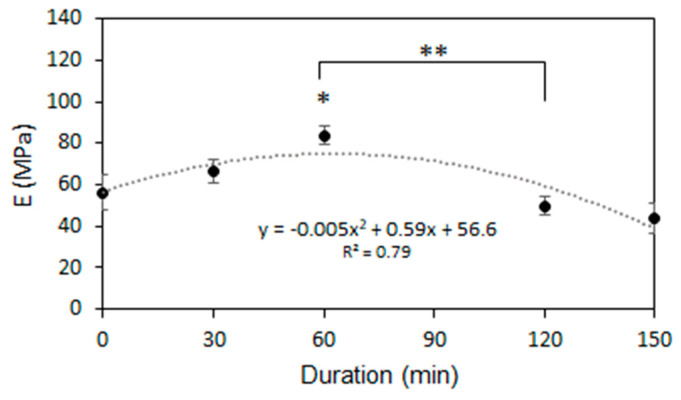
The effects of O_3_ treatment duration on PCL scaffold mechanical strength, (*) indicates a statistically significant difference compared with the untreated group (0 min), (**) indicates a statistically significant difference between groups, *p* < 0.05.

The O_3_ treatment had a non-linear effect to the mechanical strength of the scaffolds (Figure 6). Young’s Modulus appeared to increase during the 60 min of treatment from 56.2 ± 8.7 (unmodified scaffold) MPa to 83.6 ± 4.3 MPa (60 min sample). After 120 min or more, Young’s Modulus began to decrease and reached 43.5 ± 7.3 MPa (150 min), which was lower than the initial strength. Rediguieri et al. (2016) analyzed the effect of sterilization by O_3_ gas on the mechanical properties of Poly (lactic-co-glycolic acid) (PLGA) and observed the same overall trend in Young’s Modulus, although the polymer and the treatment method did not match ours exactly [24]. Many authors state that crystallinity is one of the main factors that influences the mechanical properties of the polymer [61,62]; therefore, the increased amount of amorphous regions that occurred during short-term O_3_ treatment increase the strength of the scaffold. Further treatment beyond 60 min decreases the mechanical resistance, making the scaffold more fragile and less elastic. The decrease in mechanical properties was observed due to the scission of the polymer chains that occurred in the amorphous phase [63]. Crystalline regions in the polymer are the load-bearing elements and the scission in the amorphous phase leaves them untangled, leading to the decrease in the tensile strength, as already described by other authors [64].

Based on the morphological and mechanical analyses (Figure 3 and Figure 6), the maximum O_3_ treatment time for maintaining the fibrous morphology and mechanical properties was determined to 120 min. The 150 min-treated sample was eliminated from further investigation.

### 3.4. hMDSC Proliferation

The introduction of oxygen-containing functional groups like O–C=O, C=O, C–O, and OH on the PCL scaffold surface due to O_3_ treatment not only improves its hydrophilicity, but may also accommodate biomolecule components such as proteins and cell growth factors to make the surface more attractive for cell growth and proliferation [28,42]. We thus further tested O_3_-treated samples (0–120 min) in vitro for the proliferation of hMDSC. Furthermore, we hypothesized that ozonation can enhance binding of Insulin-like growth factor 1 (IGF-1) since it is a positively charged molecule at physiological pH, and thus, electrostatically, it will bind to the O_3_-treated electrospun scaffold surface. Such a binding belongs to the non-covalent interaction method and has been shown as superior due to an ion-ion or charge-charge interaction between opposite ionic charges [65,66]. Although noncovalent bonds are weak, and they do not uphold the immobilization effect for a long time compared with covalent bonds [66], they have a transient existence at physiological temperatures (25–37 °C), allowing optimal release kinetics of growth factors [67]. Another drawback of covalently immobilized growth factors is that there may be blockage of some receptor binding sites that could connect with cells, and in order to form a covalent bonding, chemical additives, that could be toxic for cells, have to be added [18,68].

**Figure 7:**
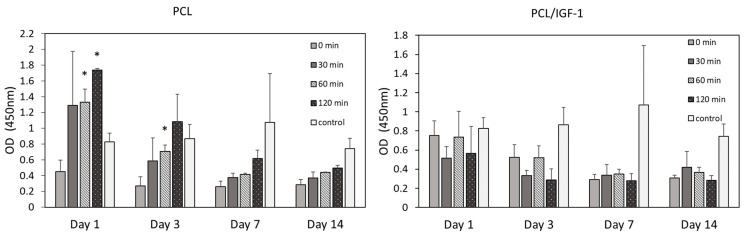
Cell proliferation determined by the CCK-8 assay (as represented by optical density at 450 nm (OD)) after 1, 3, 7, and 14 days of incubation. A PCL scaffold with different O_3_ treatment durations (0–120 min), *n* = 2; PCL/IGF-1 scaffold with different O_3_ treatment durations (0–120 min), *n* = 3; (*) indicates statistically a significant difference compared with the O_3_ untreated group (0 min), *p* < 0.05.

On PCL construct without the growth factor, the cell proliferation rate exhibited a decelerating trend in all O_3_ treatment groups throughout the 14-day period, following an exponential decrease pattern (except in the control group). Such a reduction of cell proliferation is expected for mesenchymal stem cells grown on electrospun PCL scaffolds [69], and could be related to altered cell migration and attachment to the synthetic substrate (Figure 7), although data are lacking with regard to PCL’s effect on MDSC proliferation. Nevertheless, recent studies on scaffold modifications, such as those on RGD-containing peptides or R-peptide immobilization with regard to improvement of cell adhesion and proliferation, have been published [70,71]. The data show that immobilization of RGD or R- peptides improved cell adhesion and cell-matrix interaction, leading to better cell survival and growth. Moreover, carboxyl group introduction on the surface of the scaffold can stimulate cell proliferation as well [72]. We observed that O_3_ treatment and prolongation of its duration had a positive effect on cell proliferation (Figure 7). For example, after day 1, the 120 min O_3_-treated sample resulted in a significantly higher proliferation (1.7 ± 0.02), which was 3.9 times higher compared with the untreated sample (0.45 ± 0.15). However, after 14 days, this difference diminished, but was still almost twofold higher (0.5 ± 0.03 vs. 0.3 ± 0.06). This indicates that the O_3_ treatment provided a more favorable environment for cell proliferation due to the increased hydrophilicity and water-absorption capacity. Interestingly, in the control cell group, proliferation went slightly up until day 7, but at day 14 it also decreased. No statistical difference in cell proliferation was detected in the control group at any time.

In contrast, on the PCL construct with IGF-1 (PCL/IGF-1) (Figure 7), the cells showed slower proliferation already at day 1 and day 3, compared with PCL constructs without IGF-1 in all O_3_ treatment groups, except the non-treated (0 min) group. At a later time (day 7 and day 14), proliferation was slightly more reduced in the 120 min treatment group compared with that observed on PCL constructs without IGF-1. The same proliferation pattern was observed in the control group as before.

**Figure 8:**
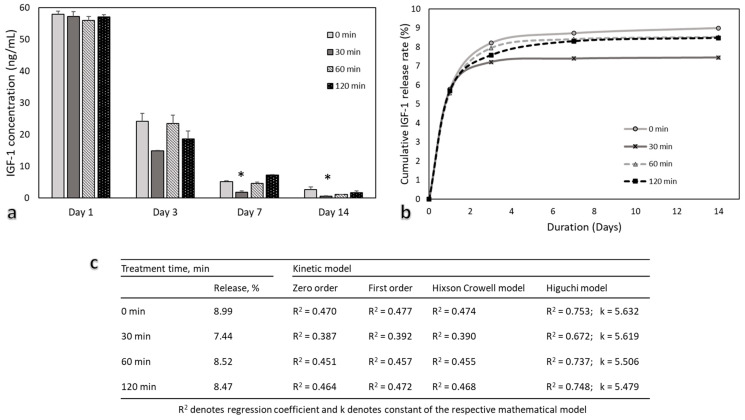
The measurement of IGF-1 content in the CM after 1, 3, 7, and 14 days. (**a**) PCL/IGF-1 scaffold with different O_3_ treatment durations (0–120 min), *n* = 3; (**b**) Cumulative IGF-1 release profile with different O_3_ treatment durations (0–120 min); (**c**) Kinetic analysis of IGF-1 with various kinetic models; (*) indicates statistically a significant difference compared with the O_3_ untreated group (0 min), *p* < 0.05.

The measurement of IGF-1 content in the CM was compared between the PCL/IGF-1 constructs with different O_3_ treatment times at day 1, 3, 7, and 14. There are two stages according to release profiles—an initial burst and a slow, sustained release (Figure 8b). The first stage occurred within the first day with 5.6–5.8% IGF-1 being released. Furthermore, IGF-1 release over the first day dominated the overall profile. Many studies have reported similar burst releases of growth factor from polymeric scaffolds occurring within the first day [32,73]. The O_3_ treatment did not affect the release of IGF-1 at day 1 or day 3 (Figure 8). Interestingly, a lower expression of IGF-1 was detected in CM when the PCL/IGF-1 construct was treated with ozone for 30 min and, in total, it consisted of only 7.44% (~74 ng) of the total amount of the incorporated protein (1000 ng) (Figure 8c). Higher levels of protein release were detected in untreated (8.99%) and 60–120 min treated PCL/IGF-1 constructs (8.52–8.47%). However, total release of IGF-1 in treated constructs was only 8.5%. The majority of IGF-1 was not released, demonstrating that there was a strong interaction between IGF-1 and the scaffold, also indicating that IGF-1 release is dependent on the initial concentration as described elsewhere [74]. Despite the fact that ionic interactions contributed to the binding, the high ionic strength did not completely eliminate IGF-1 adsorption, suggesting that there may be other non-covalent interactions. For such a system, this is a typical profile, while a slower release is usually seen at later time points [32].

To fully understand the release kinetics of IGF-1, the release data of IGF-1 were fitted using four mathematical models described in methods section. The release profiles of IGF-1 in all scaffolds achieved the best fit with the Higuchi model, as indicated by the highest value of R^2^ (Figure 8c). The Higuchi model showed that release of growth factor is a time-dependent and diffusion-controlled process, since its equation is expressed as a square root of time [75]. The release constant (k) was found by the best-fitted Higuchi model. A higher k value indicates a faster IGF-1 release. During our research, we observed that as the treatment duration increased, the k value decreased. All observations suggest that O_3_ treatment and increase of its duration could be used in order to release IGF-1 in a controllable and sustained manner.

## 4. Conclusions

A uniform PCL scaffold was fabricated using solution electrospinning, having a median fiber diameter median in the range from 0.3 to 5.1 µm and a pore size from 1.6 to 19.1 µm, depending on the PCL concentration in the solution. Surface modification using O_3_ treatment in an aqueous environment was performed, aiming to improve properties of the scaffold. ATR-FTIR spectroscopy, the number of the carboxyl group’s determination, water contact angle, and absorption capacity results confirmed a positive effect of treatment. Significant differences between FTIR spectra and the number of carboxyl groups appeared after prolonged O_3_ treatment (from 120 min to 150 min), indicating the occurrence of oxygen-containing functional groups. Incorporation of functional groups improved the hydrophilicity of the scaffolds: the water contact angle was reduced by 27% and O_3_-treated scaffolds absorbed larger quantities of PBS. The crystallinity of electrospun PCL was reduced, turning it to a more amorphous structure, especially after 120 min of treatment, which in turn accelerated the biodegradation process compared with the untreated scaffold. Since growth factors are attached to scaffold via active functional groups resulting from the treatment, the breakage of polymer chains may lead to the diffusion-driven release of the incorporated growth factors from the scaffolds.

The optimal O_3_ treatment is important. An overly long treatment may damage scaffold morphology and mechanical properties. The treatment caused the median fiber diameter to increase to 2.7 µm and 2.9 µm, (120, 150 min) compared with the untreated 2.05 µm. Furthermore, the exposure to O_3_ for more than 120 min resulted in partially fractured fiber areas, resulting in decreased mechanical properties.

O_3_ treatment and prolongation of its duration had a positive effect on hMDSC proliferation, keeping cells viable for up to 14 days. After the immobilization of IGF-1 growth factor, initial proliferation of cells on PCL/IGF-1 scaffold was slower compared with the pure PCL construct, while the growth equalized after 7 days. The IGF release profile has two distinct stages: an initial burst and a slow, sustained release. The burst release of 5.6–5.8% IGF-1 occurred within the first day. The majority of IGF-1 was not released, demonstrating that there was a strong interaction between IGF-1 and the scaffold. Such release kinetics were approximated by the Higuchi model, which suggested that the release constant (k) decreased with increasing treatment duration, indicating a slower release of IGF-1.

This study proved that O_3_ treatment in aqueous environments offers an inexpensive and effective way of ensuring modification of PCL scaffolds, aiming to improve hydrophilicity and the presence of functional groups required to incorporate a growth factor for cell proliferation enhancement. Further development and testing of this technique, such as synergetic effects ozonolysis and photolysis, and validation in vivo may be explored in order render it applicable for tissue-engineering purposes.

## Figures and Tables

**Table 1:** Main parameters of PCL solution electrospinning.

PCL concentration, % *w*/*v*	10	15	20	25
Flow rate, mL/h	2.1	2.1	2.3	2.3
Tip to collector distance, cm	11	11	11	14
Voltage, kV	12	12	14	22
